# Electrically Conductive CNT Composites at Loadings below Theoretical Percolation Values

**DOI:** 10.3390/nano9040491

**Published:** 2019-03-29

**Authors:** Brian Earp, Joseph Simpson, Jonathan Phillips, Dragoslav Grbovic, Stephen Vidmar, Jacob McCarthy, Claudia C. Luhrs

**Affiliations:** 1 Department of Mechanical and Aerospace Engineering, Naval Postgraduate School, Monterey, CA 93943, USA; bcearp@nps.edu (B.E.); jsimpson1211@yahoo.com (J.S.); 2 Energy Academic Group, Naval Postgraduate School, Monterey, CA 93943, USA; jphillips@nps.edu; 3 Department of Physics, Naval Postgraduate School, Monterey, CA 93943, USA; dgrbovic@nps.edu; 4 NREIP Summer Interns at Naval Postgraduate School, Monterey, CA 93943, USA; stephen.vidmar5@gmail.com (S.V.); scorch47@gmail.com (J.M.)

**Keywords:** CNT composites, percolation mechanisms, conductive CNT composites, excluded volumes, CNT dispersion

## Abstract

It is well established that dramatic increases in conductivity occur upon the addition of conductive filler materials to highly resistive polymeric matrices in experimental settings. However, the mechanisms responsible for the observed behavior at low filler loadings, below theoretical percolation limits, of even high aspect ratio fillers such as carbon nanotubes (CNT) are not completely understood. In this study, conductive composites were fabricated using CNT bundles dispersed in epoxy resins at diverse loadings, using different dispersion and curing protocols. Based on electron microscopy observation of the CNTs strands distribution in the polymeric matrices and the corresponding electrical conductivities of those specimens, we concluded that no single electron transfer model can accurately explain the conductive behavior for all the loading values. We propose the existence of two different conductive mechanisms; one that exists close to the percolation limit, from ‘low loadings’ to higher CNT contents (CNT % wt > 0.1) and a second for ‘extremely low loadings’, near the percolation threshold (CNT % wt < 0.1). The high conductivity observed for composites at low CNT loading values can be explained by the existence of a percolative CNT network that coexists with micron size regions of non-conductive material. In contrast, samples with extremely low CNT loading values, which present no connectivity or close proximity between CNT bundles, show an electrical conductivity characterized by a current/voltage dependence. Data suggests that at these loadings, conduction may occur via a material breakdown mechanism, similar to dielectric breakdown in a capacitor. The lessons learned from the data gathered in here could guide future experimental research aimed to control the conductivity of CNT composites.

## 1. Introduction

Electrically conductive CNT composites are in high demand due to the broad range of applications that they could enable; from anti-static materials used in fuel tanks, housing materials and containers, aerospace structures and electromagnetic interference shielding systems, to sensors and conductors used as metal replacements and thermoelectric materials among others [1,2,3,4]. Given the non-conductive nature of most polymeric matrices, the traditional approach to produce conductive composites has been the addition of conductive fillers. CNTs provide not only a conductive phase to be introduced in the polymeric matrix of the composite, they also present high aspect ratios that promote conduction at lower loading than spherical or irregularly shaped fillers. However, despite the existence of numerous publications in regard to CNT composites and their properties, reports aiming to explain the mechanisms that dominate the electrical conductivity at extremely low loading CNT values (% wt CNT below 0.1%) are scarce. It is generally agreed that the high conductivity observed even at low loadings of the conductive phase is related to (i) percolation, that is, to the formation of continuous strings of conductive material spanning in a non-conductive matrix material [5,6,7,8], (ii) a combined percolation-tunneling effect that allows electrons to hop from one conductive particle to another in close proximity [5,7,9], and/or (iii) the existence of excluded volumes in the matrix where no conductive material exists [10]. In standard percolation theory [11], based on conduction paths forming along strings of randomly placed conductive spheres in a non-conductive matrix, conductivity is only enhanced at relatively high loadings. Specifically, only if the loading of the conductive phase exceeds ~9–18%, the so-called ‘percolation threshold’, an enhancement of conductivity can be observed [12,13]. The influence of non-spherical conductive inclusions, particularly high aspect ratio fillers such as CNT, and how those disperse in the matrix have been scrutinized by diverse research groups, with reports of conductive samples (σ = 10^−2^ Sm^−1^) achieved at loadings that decrease the percolation threshold to values as low as 0.1% vol. [5,6,9,14,15,16]. Other reports point to the generation of CNT conductive composites at even lower loadings [2,14], some with threshold values reaching 0.0025% wt [7], where factors such as the CNT high aspect ratio and alignment, processing conditions, and temperature, play an important role in the conductive network formation. In the present work it is argued that a model consistent with all observations of conductivity over a range of CNT loadings must include consideration of: (i) properties of the conductive phase, (ii) impact of processing protocols on dispersion, (iii) the effective percolation volume of high aspect ratio conductors, (iv) excluded volumes, and (v) mechanisms of dielectric electron transport over short distances.

Detailed studies of the impact of production parameters on the electrical resistivity of epoxy–CNT composites are presented in the following sections. In particular, we report resistivity values and analyze the impact that (1) CNT concentration, (2) bundle size that results from the pre-mix grinding step, (3) use of beads to aid dispersion, (4) number of cycles used for the mixing protocol, and (5) curing processes have on the microstructural features of the specimens and their correlation with electrical properties. Based on these observations, qualitative mechanisms, employing only a few simple concepts, are developed to explain all observations in a consistent fashion.

## 2. Materials and Methods

### 2.1. Materials

The CNTs used for this study, Miralon Pulp^®^ from Nanocomp Technologies, Inc. (Parent organization: Huntsman Corporation, Merrimack, NH, USA) were porous bundles of multiwall, not functionalized nanotubes. The CNTs were produced in large sheets using a chemical vapor deposition process with an iron catalyst and then made into a pulp using a Hollander Beater and industrial burr mill, resulting in bundles of approximately 0.05 mm in diameter and 1mm in length, as described in the company’s webpage [17]. CNTs from various Nanocomp-generated lots were used during the course of this study, however the data used for direct comparisons were obtained using CNTs from the same lot. The polymeric resin used was LOCTITE EA9396 AERO epoxy paste adhesive (Hysol EA9396, Henkel Corporation, Dusseldorf, Germany). This epoxy is a low viscosity two part system that is mixed in ratio of 100 Part A to 30 Part B in accordance with manufacturer’s guidance [18]. The epoxy can be cured using one of two methods: (a) room temperature cure (3 to 5 days at 25 degrees Celsius) or (b) accelerated cure (1 h at 66 degrees Celsius). Epoxy composites containing CNT bundles in weight percentages between 0.014 and 2% wt were produced and analyzed using the techniques and protocols mentioned in the following sections.

### 2.2. Characterization Techniques

Scanning electron microscopy (SEM) was the technique employed to analyze the microstructural features of CNT pulp samples and epoxy–CNT composites. We used a Zeiss Neon 40 (Carl Zeiss Inc., Thornwood, NY, USA) field emission SEM operating between 1 and 20 kV. This instrument is coupled with an INCA Energy 250 Energy Dispersive X-ray microanalysis system with analytical drift detector. A FEI Tecnai Osiris Scanning Transmission Electron Microscope (STEM) (Hillsboro, OR, USA) operating at 200 kV was employed to acquire the high-resolution TEM (HRTEM) image presented. In order to be studied by TEM, thin composite specimens were scratched at the surface using an X-Acto knife and placed in a holey carbon copper grid. A Netzsch STA 449 Jupiter F3 simultaneous thermal analyzer (Selb, Germany) was employed to perform temperature programmed oxidation (TPO) of the CNT samples to determine the amount of Fe catalyst contained in each lot. The samples were burned off from room temperature to 900 degrees Celsius in a 20% O_2_ and 80% N_2_ atmosphere. The CNT bundle sizes and porosity were observed using a Nikon Epiphot 200 Optical Microscope (Tokyo, Japan). Electrical resistivity measurements were conducted using a 2400 Keithley Source Meter (Beaverton, OR, USA) or Harrison 6110A DC Power Supply (Palo Alto, CA, USA) and a multimeter.

### 2.3. Fabrication Protocols

CNTs were introduced into EA9396 epoxy using various protocols that varied (1) CNT concentration, (2) bundle size that results from the pre-mix grinding step, (3) use or absence of 3 mm glass beads to aid dispersion, (4) number of cycles used for the mixing protocol, and (5) the temperature/time employed to cure the specimens, as described below. For all mixing methods, Part A, EA9396 epoxy resin was measured and CNTs were added to it based on calculation of desired weight percentage using total expected weight of Part A epoxy resin, Part B epoxy hardener, and CNTs. All samples were mixed using a FlackTek, Inc. Speed Mixer Model DAC150.1 FV2-K. The mixing process typically consisted of a two cycles initial lower speed mix followed by 3 to 12 higher speed mixes of one minute in length. The epoxy–CNT samples were allowed to cool to prevent excessive heat buildup between mixing cycles. Following speed mixer cycles, Part B epoxy hardener was added to the Part A epoxy resin and CNT mixture and hand mixed for 5 min.

After mixing, a thin layer of the epoxy–CNT mixture was placed onto a four-point test board. The testing boards consisted of eight testing locations that each had four electrodes. Thus, for each variable under study we extracted between four and eight readings. The inner electrodes were 1 cm apart for each testing location. The test boards were prepared by placing two pieces of tape, 1 cm apart, along the edges of the board to allow the epoxy–CNT mixture to be laid down before curing. A glass slide was used to level the epoxy composite to create a relatively uniform thickness along the 1cm wide sample strip that ran the length of the test board. The test boards with the epoxy–CNT sample were then cured via two different methods based on the epoxy manufacturer’s guidelines. The primary curing method was an accelerated cure in a Lindberg Blue furnace at 66 degrees Celsius for one hour and the secondary method was a slow cure at room temperature (25 degrees Celsius) for three to five days.

### 2.4. Electrical Resistivity Measurements

Following the curing process, electrical measurements of the epoxy–CNT composites were performed as follows: current was passed through the outer electrodes of the test station and voltage drop across the inner electrodes of the test station was measured using a digital multimeter. The applied current typically ranged between 5 µA and 100 µA, however currents up to 2000 µA were used for some parts of the study. The epoxy–CNT composite’s sample thickness and area were measured and resistivity was calculated using the following formula:

$$\rho =\;\frac{RA}{L}$$

where *ρ* is resistivity, *R* is resistance (Ohms), *L* is sample length (1 cm in our case), and *A* is cross section area of the measured sample (1 cm × sample thickness), thus simplifying the resistivity equation to:

$$\rho =\;\frac{R}{t}$$

where *t* is sample thickness. The calculated resistivity values were used for data analysis.

## 3. Results

**Figure 1:**
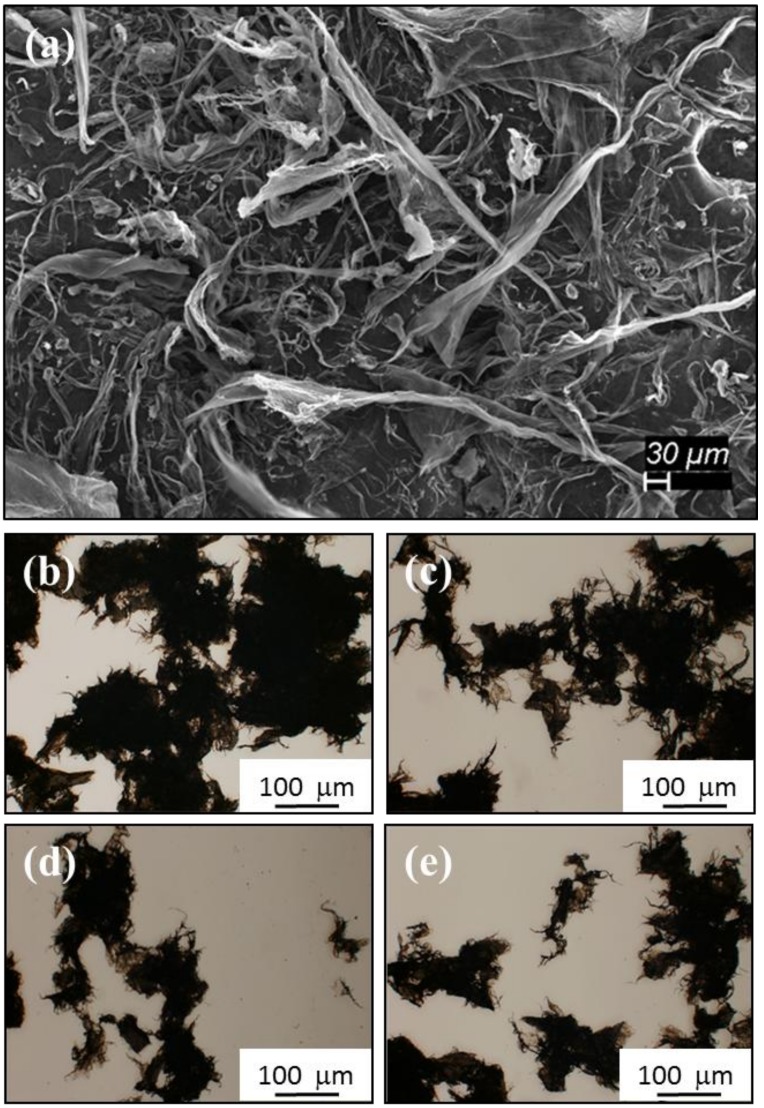
(**a**) Scanning electron microscopy (SEM) image of carbon nanotubes (CNT) pulp as received. (**b**–**e**) OM images of CNT bundles of diverse sizes generated by different cutting and grinding conditions: Grind 1,2,3, and 4 respectively.

The SEM image in Figure 1 reveals the microstructural features of the CNT pulp bundles as received from manufacturer and prior to mixing with epoxy to form the epoxy–CNT composites. In particular, Figure 1a shows an SEM image at 100× magnification of Lot 2, Grind 1 CNT pulp. As observed, the bundles consist of multiple entangled CNTs. The bundles showed no directionality or evidence of alignment. The Fe catalyst particles are encountered throughout the sample with sizes in the nanometer scale. The production process followed by the beater/mill step can generate CNT bundles of diverse sizes. For this work we will denominate the later as Grind 1, 2, 3, and 4. Grind 1 was the largest pulp size used in this study. Figure 1b–e present optical microscopy (OM) images of Grinds 1, 2, 3, and 4 for comparison. In all cases, the bundles present multiple ramifications and strands that expand in random directions. The central part of the bundles presents more mass than the edges. As the grind number increases the bundles acquire higher aspect ratios, but also break up into smaller bundles, while maintaining sizes in the micron scale. The geometry and distribution of the strands is greatly modified once introduced into the epoxy resin, as will be evident in the results that follow.

**Figure 2:**
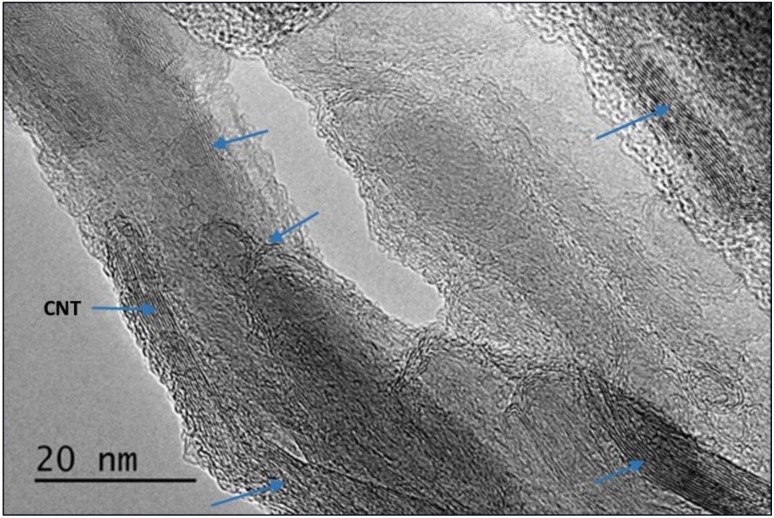
HRTEM micrograph of the CNT–polymer structure showing the nanotube location (marked by arrows) in the composite cluster. Bare polymer was identified in the center of the particulates.

Simultaneous thermal analysis (STA) using TPO conditions revealed that the CNT pulp samples contained between 11% and 14% wt of Fe, depending on the lot under study. Figure 2 presents an HRTEM image of a polymeric sample containing 0.014% wt of CNTs. The image provides a good illustration of the small space occupied by individual CNTs relative to the epoxy matrix. It also shows CNTs occupying sections of the composite structure along regions completely depleted of CNTs, the later found normally ‘encased’ by the CNT strands. These regions, where no CNTs are found, are referred as excluded zones or excluded volumes.

### 3.1. CNT Concentration in the Epoxy Composite

**Figure 3:**
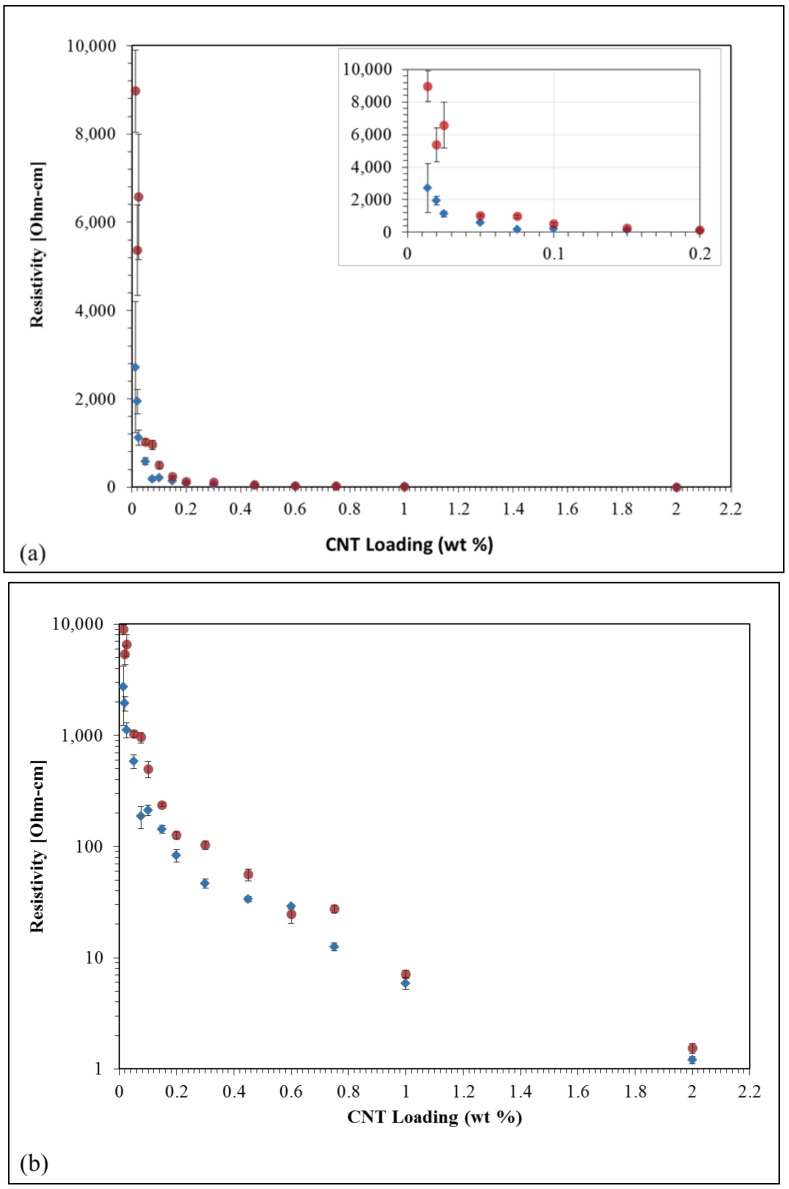
Trends in composite resistivity as function of CNT loading. Blue: lot 1, Red: lot 2. (**a**) Linear scale with inset: Composites at extremely low loading values and (**b**) logarithmic scale.

Figure 3 displays resistivity of the epoxy composite samples as a function of CNT loadings from 0.014 to 2% wt in both linear and logarithmic scales. While samples from different lots, as described in the experimental section, were used throughout this overall study, the resistivity vs. CNT loading graph presented in Figure 3 only used two of those to show the lot-to-lot variability and general trends. The resistivity trends found are consistent with other studies [5,6,7,9], particularly in terms of a dramatic increase in conductivity, clearly visible in Figure 3 inset, as the content of CNT is increased beyond a tipping point. For example, composites containing 0.014% wt CNTs show resistivity on the order of 3 × 10^3^ Ohm-cm (for lot 1, 9 × 10^3^ Ohm-cm for lot 2), whereas specimens containing 0.025% wt CNTs were found to have approximately one order of magnitude less resistivity. At the high end of this loading range, for loadings close to 0.2% wt CNT and above, the resistivity is between 2 and 3 orders of magnitude lower than that of the epoxy resin with 0.014% wt CNTs. It is important to note that even the 0.014% wt CNT samples have a resistivity approximately eleven orders of magnitude lower than the manufacturer reported resistivity of the epoxy without CNTs [18].

It is worth noting that the standard deviation for the conductivity of a single concentration (taken from a minimum of 4 to 8 sample points as described in the experimental method section) for the same lot is much smaller than that observed between lots, however, changes in the CNT concentration in the epoxy composites produce general trends that are observable and reproducible in multiple CNT lots. Values of what have come to be known as the percolation threshold and percolation limits seem to be within similar orders of magnitude for all lots. Given that the composite samples were produced, cured and tested with identical experimental conditions, we can imply that only the differences between CNT fabrication conditions at the CNT manufacturing sites are responsible for the lot-to-lot variability seen in Figure 3.

Given that CNT concentrations of 0.014% and 0.75% wt in the epoxy composite present resistivity values of different orders of magnitude, those two concentrations were employed for the other sections of the study. In order to analyze other factors that influence the electrical behavior of the composites we conducted experiments that aimed to determine how samples’ microstructure and electrical properties were affected by (i) CNT bundle size, (ii) use of beads during mixing to aid dispersion, (iii) mixing protocol in terms of the number of dispersion cycles, and (iv) variations in the curing process

### 3.2. Effects of the Use of Glass Beads to Aid Dispersion

**Figure 4:**
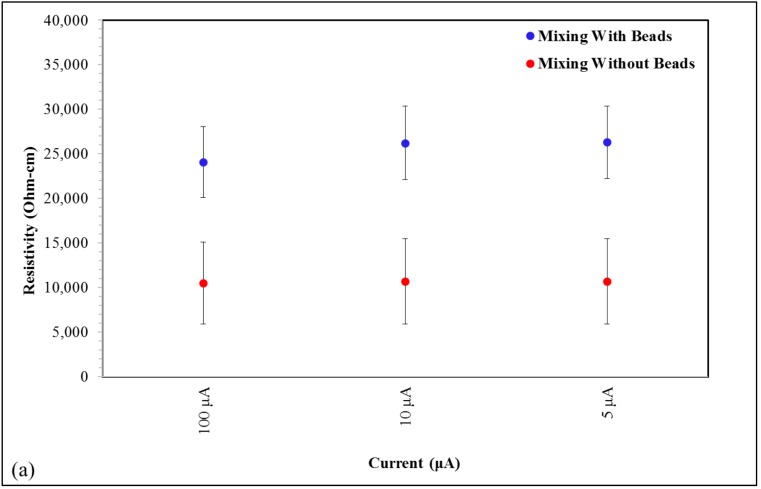

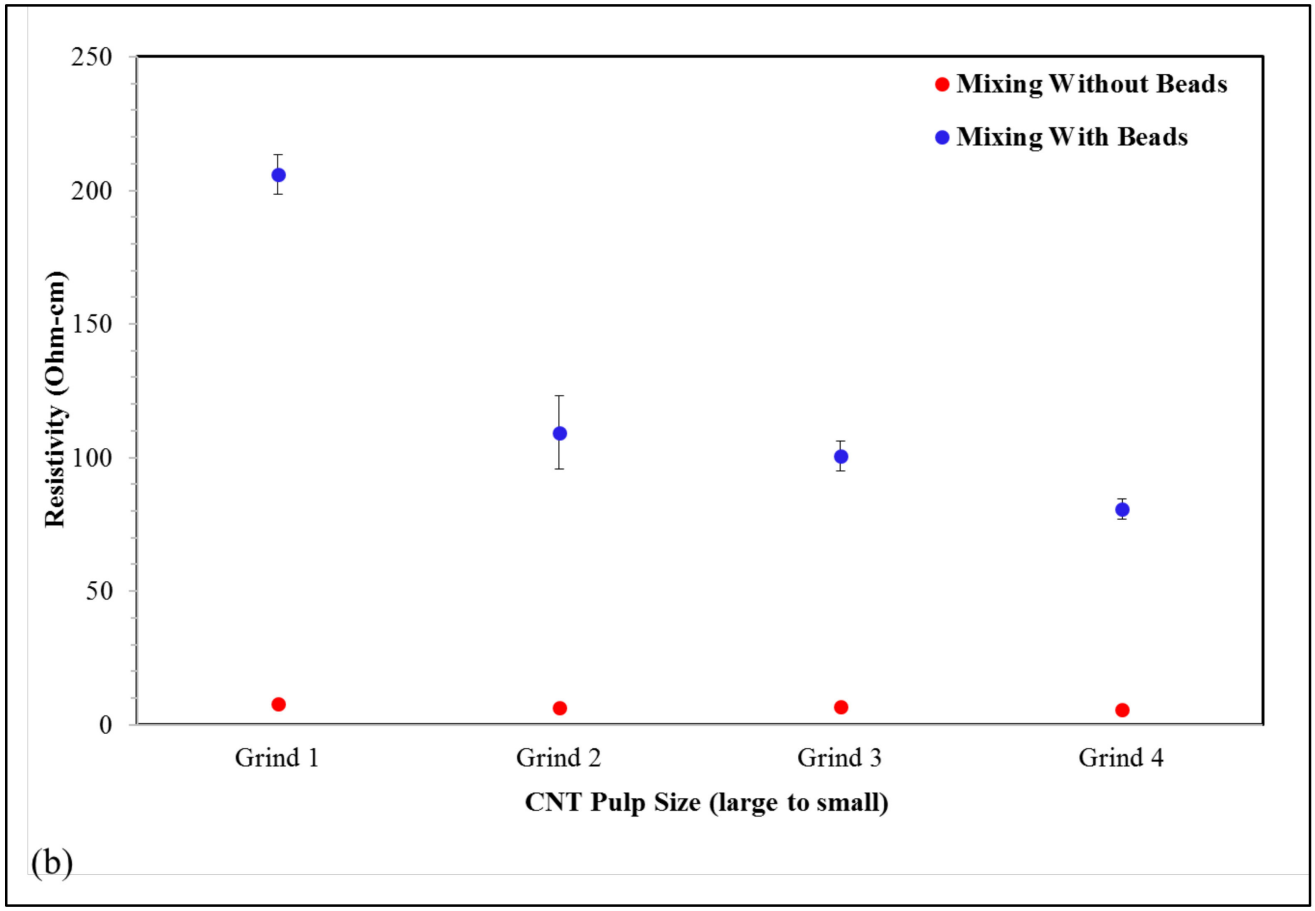
Effect of the use of beads during the CNT and epoxy resin mixing process for (**a**) 0.014% and (**b**) 0.75% wt CNT epoxy composites. Figure 4a shows also the measured resistivity as a function of applied current (5 µA, 10 µA, and 100 µA) at 0.014% wt CNT loading while grind sizes effect on samples containing 0.75% wt CNT is included in Figure 4b.

Figure 4 shows the impact that the use of beads and bundle size have on the resistivity of the specimens. As shown in Figure 4a,b, the inclusion of beads during the mixing process, for both loadings (0.014% wt and 0.75% wt), significantly increases electrical resistivity. The inclusion of beads in the mixing process was found to have a more profound impact on resistivity than any other process parameter. The presence of beads was found to increase resistivity by as much as 2.5× relative to the same process without beads for samples with 0.014% wt CNTs while in samples with higher CNT loadings (ca. 0.75% wt), the effect is more substantial, resulting in resistivity increasing 1 to 2 orders of magnitude (Figure 4).

### 3.3. Pre-Mix CNT Grinding Size

The grinding size seems to provide a certain level of variability between the 0.75% wt CNT composite samples; however, it only becomes significant when glass beads are employed during the dispersion of CNTs in the resin, with larger grinding size increasing resistivity by a 2× factor. Of note, the general trend is that the smaller pulp size CNT grind results in a lower resistivity value. The grinding size of the CNT pulp in the resistivity of the composite samples with 0.014% wt CNT was found to be within the variability of a single sample and not as impactful as any of the other parameters previously mentioned or the current dependence that can be seen in Figure 4a.

These effects are attributed to the beads leading to a more even distribution of CNTs in the matrix. In the absence of beads it is shown (below) that the CNT distribution is limited to relatively small volumes, ‘zones’, within the matrix. That is, in the absence of intense mixing the matrix is more inhomogeneous with zones of high concentrations of CNTs and other zones without any CNTs. Those will play a significant role in the generation of excluded volumes, as will be later discussed.

### 3.4. Effect of Number of Dispersion Cycles Used to Combine CNT and Epoxy Resin

**Figure 5:**
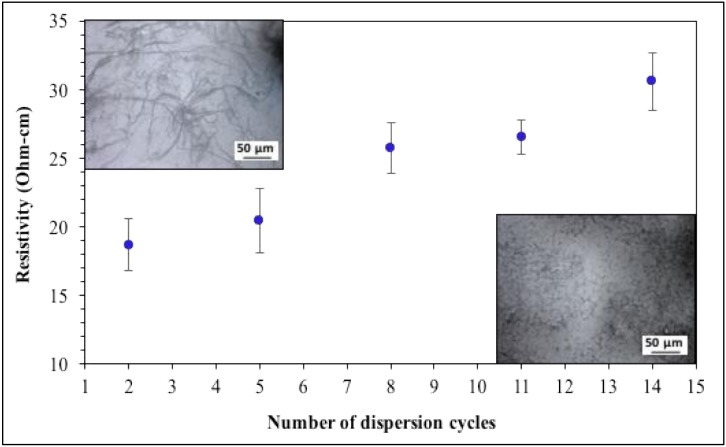
Effect of number of dispersion cycles employed during the fabrication of composites with 0.75% wt CNT on the sample resistivity. The OM inset images correspond to 2 and 14 cycles (left and right respectively). The scale bar represents 50 µm.

For this section of the study, samples from the same lot of CNTs were mixed with epoxy resin using 0.75% wt CNT loading, but a different number of dispersion cycles before adding the hardener and curing at 66 degrees Celsius for one hour. The resistivity values generated from such trials are presented in Figure 5.

The impact of increasing the number of dispersion cycles is not as significant as the use or absence of beads; however, it does change resistivity values, particularly for the higher loading (0.75% wt samples). The effect of using prolonged dispersion protocols in samples with 0.014% wt CNT (not included) was minimal. That is, the contrast between using loadings of 0.75% or 0.014% wt determines how much the dispersion cycles will affect the sample. For 0.75% wt highly dispersed specimens the excluded zones get destroyed, resulting in breakup of conductive networks. For extremely low loadings (0.014% wt) it is believed the CNT are nearly homogenously distributed but incapable of forming continuous networks, independent of mixing protocol including use/nonuse of beads, number of dispersion cycles, and even pulp characteristics. The OM images presented in Figure 5 support the postulate that highly dispersed CNTs have thinner strands and do not form continuous networks.

### 3.5. Effect of Curing Temperature/Time

**Figure 6:**
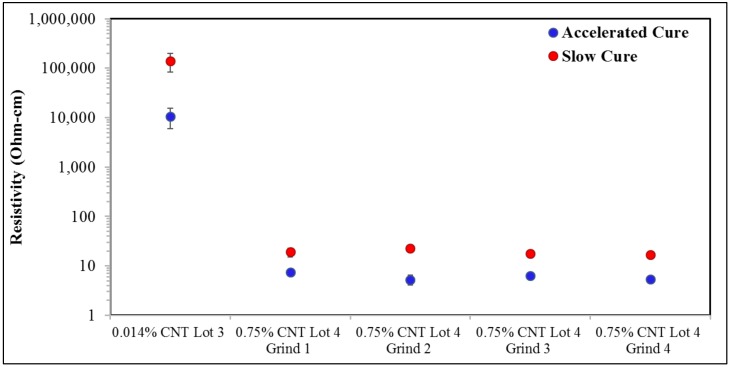
Impact of curing time/temperature on composite resistivity. All samples were measured at 5 µA.

In Figure 6, for the extremely low CNT loadings (0.014% wt), the resistivity of samples cured at 25 degrees Celsius for approximately 120 h is an order of magnitude larger than samples cured via an accelerated process of 1 h at 66 degrees Celsius. The curing time has a similar, but smaller magnitude, impact on the resistivity for samples containing 0.75% wt CNTs. An accelerated cure at 66 degrees Celsius for one hour decreased resistivity by about a factor of 3× relative to the 25 degrees Celsius slow cure. As shown in Figure 6, this impact of curing time, magnitude and direction, is virtually the same for 0.75% wt CNT samples of all ‘grinds’.

### 3.6. Applied Current Impact

**Figure 7:**
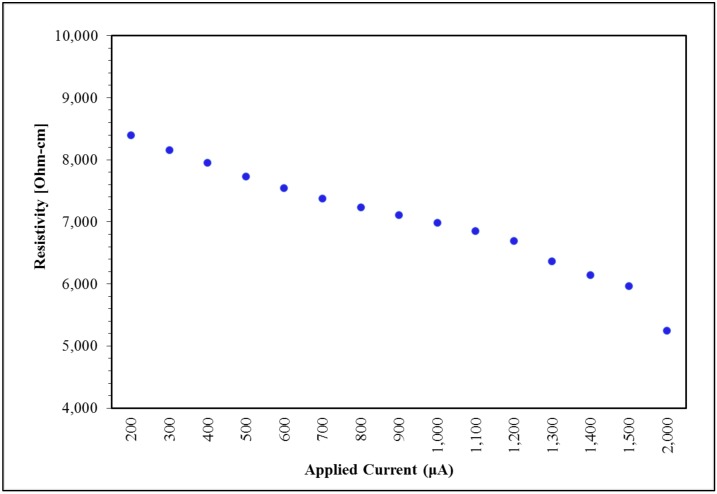
Applied current impact on resistivity at 0.014% wt CNT Loading.

Based on observations during the initial parts of this study that extremely low loading samples were impacted by current changes, a closer evaluation of 0.014% wt samples was conducted at various currents, making it to be the sixth variable studied. Figure 7 is a plot of resistivity vs. current for a 0.014% wt sample that was subjected to initial mixing and nine subsequent mixing cycles. There is an evident lowering trend in resistivity as current is increased. The changes in resistivity observed when measured at 200 or at 2000 µA are within the same order of magnitude, nonetheless they are greater than the standard deviation taken from the eight sections of the board for the 0.014% wt CNT specimen. No such trend was observed for any of the 0.75% wt CNT specimens.

### 3.7. SEM Imaging of Epoxy–CNT Composite Microstructural Features

**Figure 8:**
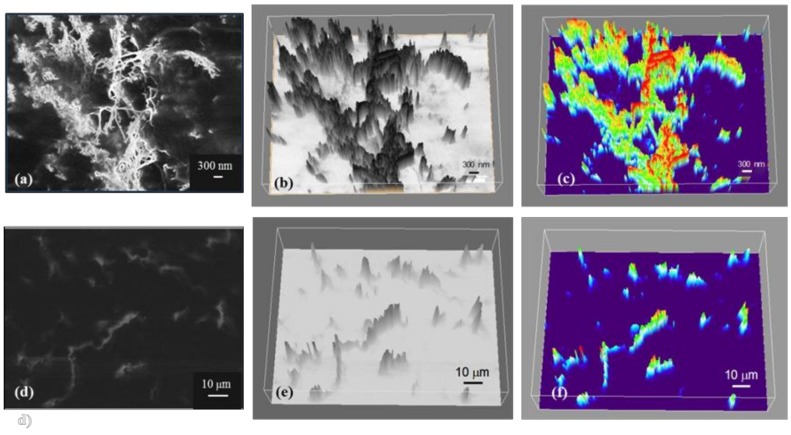
Top: 0.75% wt CNT composite showing the CNT forming a network within the epoxy matrix. (**a**) Original SEM secondary electron image; (**b**) 3D surface plot generated from greyscale image and (**c**) 3D surface plot ‘thermal’ image. Bottom: 0.014% wt CNT composite structure; (**d**) SEM micrograph showing unconnected strings on CNTs; (**e**) 3D surface plot greyscale image and (**f**) 3D surface ‘thermal’ plot.

In an attempt to gain a better understanding of the conduction mechanisms in the epoxy–CNT composites at 0.75% and 0.014% wt CNT loadings, the specimens were polished and analyzed using SEM. The secondary electron images generated at 20 KV are presented in Figure 8d, respectively. The location of the CNTs within the matrix and the density of the CNT strands seem quite different for each concentration: the images acquired for samples loaded with 0.75% wt CNTs show a network of connected or in very close proximity CNTs which expands in all directions, clearly forming a web of CNTs at the submicron scale that alternates with CNT-free zones. In contrast, the strands of nanotubes in samples loaded with only 0.014% wt CNTs expand in different directions and most of them are isolated from each other by distances that span from a few to tens of microns. Only the brightest spots in the SEM images are in focus, regions that seem out of focus or of other tones of grey correspond to strands located under the surface, embedded in the matrix.

The differences in the structures with different CNT loadings are also evident when observing Figure 8b,e, which correspond to the 3D greyscale surface plot of the samples constructed using Image J software [19] from the original SEM images. The surface plots attempt to represent the tridimensional nature of the CNT networks based on the tones of grey found in the SEM experimental images. Figure 8c,f present the simulated ‘thermal’ images, also generated from the original SEM micrograph using Image J. On those, the red sections represent CNTs located at the surface of the samples, the green/yellow sections represent CNT bundles/strands that are located below the surface, and the purple sections represent bare epoxy matrix. The SEM images and the pictograms illustrate one of the main findings of the study: in samples with extremely low concentrations near the observed percolation threshold (0.014% wt CNTs), even with the high aspect ratio of the filler, the CNTs are not in close proximity to allow electrons to freely hop between stands, in contrast, samples with low concentrations (0.75% wt CNTs) the CNTs high aspect ratio and the existence of zones depleted of CNTs allow for a conductive path to be formed at effective concentrations below those predicted by classic percolation theory.

Based on the physical characteristics observed for samples with ‘low loadings’ (>0.1) and ‘extremely low loadings’ (<0.1), the resistivity data presented in previous sections regarding effects of dispersion cycles, use of beads, and curing conditions can be easily understood: for the low CNT concentration samples, a ‘percolating’ conductive network forms when the matrix is inhomogeneous, specifically, when the CNTs are confined to only a fraction of the total volume. Any process, including the use of beads, or an increase in the number of dispersion cycles, that increases the homogeneity of the CNT dispersion by breaking down the ‘excluded volumes’, invariably increases the distance between conductive strands, hence reducing the connectivity and concomitantly the macroscopic conductivity. In contrast, samples that contain extremely low CNT loadings (0.014% wt) are already isolated from each other, present minimal effects due to higher levels of dispersion and favor the conduction of electrons when higher currents are applied. Moreover, the specimens with extremely low CNT concentrations tend to have higher standard deviations, thus, the effects of other parameters tend to fall within the expected test board location-to-location variability.

Finding an explanation for the observed effect of curing time/temperature presents a bigger challenge. One hypothesis is linked to the nature of the polymer-CNT interactions: specifically, strong interfacial links reduce the electrical conductivity of the individual CNT by (i) reducing the number of free electrons, and (ii) creating scattering centers. Moreover, it is believed that the strength of such CNT–polymer interactions might be a consequence of the solidification process. That is, polymers cured rapidly (high temperature cure) are more likely to homogeneously nucleate and simply ‘trap’ the CNTs, while slow curing (room temperature cure) enables the CNTs to serve as nucleation sites, promoting stronger CNT–polymer connections. Designing experiments to test such hypothesis will certainly be the recommended next step.

Other factors that deserve further investigation include studies regarding strand density, which should be optimized to reduce the appearance of hot spots and, given the 0.75% wt CNT composite microstructural configuration, studies that determine if the existing network configurations could lead to the formation of intervening circular loops that could limit the end-to-end current transport.

## 4. Discussion

The primary objective of this work, developing a practical road map for controlling CNT composites’ conductivity, requires a detailed understanding of the effect of all parameters, including material, CNT loading, preparation protocols, etc. on morphology and conductivity. The results obtained demonstrate that for low loadings (>0.1% wt CNT) trends in resistivity as a function of loading and production protocols can be well explained using existing models. However, the present results suggest a new model for conductivity, and impact of production protocol, for extremely low CNT loading (<0.1% wt CNT). Finally, a heuristic for obtaining high conductivity CNT-containing composites is presented.

### 4.1. Novel Experimental Design Features

In prior conductive composite work, the implicit assumption of the experimental design was that conductivity tracks the concentration of the conductive component as it relates to the presence of percolation networks given the filler aspect ratio or other geometrical factors and how ‘well’ dispersed those are in the polymeric matrix [8,15,20,21,22]. In the present work the experimental design reflected a different assumption: The production factors that impact resistivity are unknown. Thus, the design reflected the potential for any parameter to impact composite resistivity. In fact, the results justify this experimental design approach. The data shows that some changes in protocol change resistivity by more than an order of magnitude, whereas others have very little impact.

Another factor explicitly considered in the experimental design was CNT loading. It was hypothesized that impact of production protocol on the specimen’s resistivity would be different for low concentrations than for extremely low concentrations (<0.1% wt). In fact, this was found true. For example, mixing had a strong impact on low loading samples, whereas at extremely low concentration, CNT mixing had minimal impact.

### 4.2. Percolation

In all of the explanations advanced below, percolation through the high conductivity material in the composites, that is CNTs, is given as the main mechanism of conductivity, hence a brief review is offered. To wit: Measured ‘net’ conductivity (resistivity) arises from a connected path of high conductive filler particles in a non-conductive matrix media. Insulating matrix media is assumed to play a negligible role in the overall measured conductivity [11]. Classic percolation theory [11,12,13], assumes a spherical conductive filler. Electrons find a path from one end to the other only if there is a continuous path of filler particles from one end of the material to the other. This network of connectivity can be modelled as a ‘resistor network’ in which most of the resistance is found at the points of conductive filler contact. Although there is a ‘loading’ at which percolation begins (~9–18% for spheres) [12,13] the net resistance is also a function of the total filler loading. For percolation networks, as with all resistor networks, the larger the number of ‘paths’ the smaller the net resistance (e.g., resistors in a parallel network). As discussed below, for high aspect ratio conductive filler (e.g., CNTs) percolation begins at far lower loading than it does for spheres, but the basic mechanism is similar [2,7,14,16]. There must be a complete path for any percolation to occur, and the main points of resistance are found where CNTs touch. Moreover, the more paths exist, the lower the net resistance.

### 4.3. Trends Detected

#### 4.3.1. Mixing Cycles

**Figure 9:**
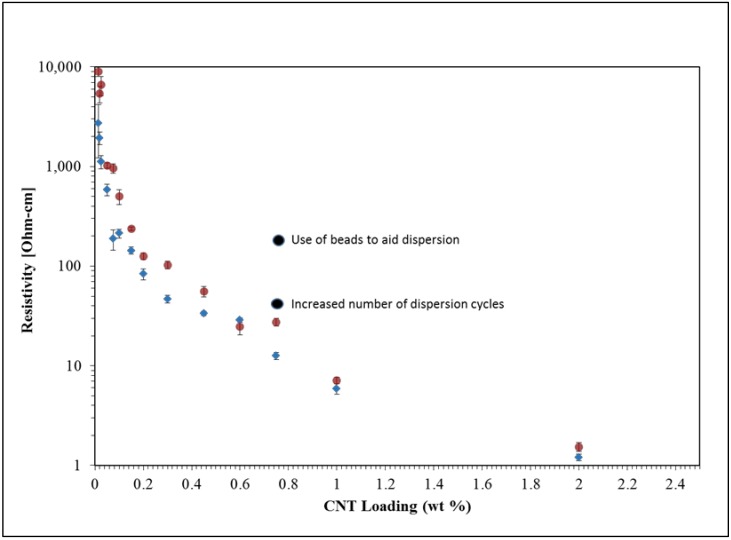
CNT epoxy composites resistivity vs. filler loading. Note the impact that CNT loading values have when compared to those observed when using dispersion protocols such as use of beads and increase number of dispersion cycles.

**Figure 10:**
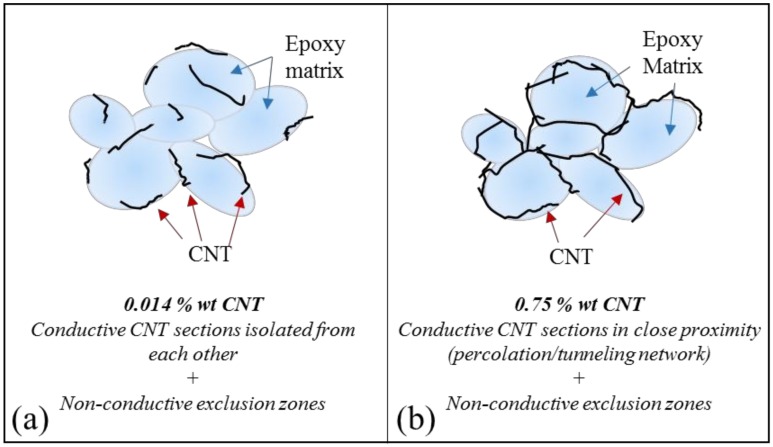
Pictograms of the CNT and epoxy distribution in composites containing (**a**) 0.014% wt CNT and (**b**) 0.075% wt CNT.

Many reports indicate that a highly dispersed filler is a required condition to achieve high conductivities, and mixing is designed to ensure homogeneous distribution of conductive filler [2,7,23]. In contrast, results of this study (Figure 5) indicate that a minimal mixing yields the lowest composite resistivity for low loading samples, while mixing has little impact on extremely low loading samples. Figure 9 reproduces the graph in Figure 3 with a data point that indicates the change in resistivity for the 0.75% wt CNT sample when increased mixing cycles are used. The negative impact of mixing on low loading samples correlates with the theory of the larger ‘effective volume’ of high aspect ratio conductive materials. Repeatedly it has been shown that even 1% volume fraction loading of high aspect ratio conductive fillers, such as CNT, increases the conductivity of composites by many orders of magnitude [14]. This is consistent with models [10] of this effect, in which it is postulated high aspect ratio conductive filler effectively ‘sweep out’ a volume far larger than the actual volume of the filler. That is, high aspect ratio filler particles effectively connect to form percolation networks over volumes far larger than their physical volume would allow in the classic model of spherical particle percolation [24]. In practice, this leads to the finding that 1% wt CNT filler [5,14] can increase conductivity via percolation as well, or better, than the same matrix materials containing 9+% volume spherical filler particles. Figure 10 presents a pictogram that might aid these concepts and how they apply to the low and extremely low loadings cases under study.

The minimal impact of mixing on extremely low loading samples is also consistent with this model. At extremely low concentrations, networks do not form even if there is no mixing. The ‘effective volume’ of the high aspect ratio CNTs is still not large enough to ensure percolation paths form even for the unmixed samples at these loadings. Thus, even if mixing does homogenize the morphology, it does not break up percolation paths since few initially existed.

Another mechanism for increasing the ability of fillers to form networks is to confine them to only a fraction of the total volume of the matrix. The volume of the matrix in which the filler is not found at any concentration is called the ‘excluded volume’ in the literature [10]. Given an unchanged total filler fraction, the existence of excluded volumes will increase the concentration of filler in all the ‘included volumes’. As illustrated elsewhere [10], this permits percolating paths of both spherical and high aspect ratio conductive filler to form at lower net loadings through connected ‘included volumes’, than would be required for homogeneous distributions.

SEM imaging conducted in this study clearly shows, uniquely, the formation of the postulated excluded zones (Figure 8). If these excluded zones are removed by vigorous mixing, the CNT percolation paths will either disappear entirely, or at a minimum become thinner and have reduced conductivity.

#### 4.3.2. Mixing with Beads

One standard method employed to increase homogeneity in composites is to use beads in high speed mixtures. As mixing increases the homogeneity of the CNT distribution, and concomitantly removes exclusion zones, the theory advanced above suggests enhanced mixing with added beads will even further reduce conductivity. This is clearly observed (Figure 4). The highest increase in resistivity observed with the use of beads with low loading CNT-epoxy composites was in fact almost 25×. Figure 9 reproduces the graph in Figure 3 with a data point that indicates the change in resistivity for the 0.75% wt CNT sample when mixing beads are used. In the case of the 0.75% wt CNT loading samples, the quantitative impact of this parameter was also influenced by the grind given the CNTs prior to loading. Clearly, some ‘grinds’ suffered a far more dramatic increase in resistance than others. Postulate: Some grinds are more difficult to homogenize by mixing/beads than others. Those more resistant to homogenization will retain more connectivity/conductivity following mixing.

The extremely low loading composites (0.014% wt) did show increased resistance with the use of beads, but it was a relatively minor (up to 2.5×) effect. This is consistent with the postulate above that at extremely low loadings few true percolation paths exist, hence mixing cannot remove them.

#### 4.3.3. Curing

Rapid curing at elevated temperature of samples clearly improved conductivity, particularly of the extremely low loading samples. Indeed, the resistivity of the slow cured, extremely low loading samples was more than a full order of magnitude higher than that of the accelerated cure samples. The change in resistivity was far less for the 0.75% wt samples, approximately as much as 3× Also, unlike the mixing case, the grinding method employed had little impact on the results. These results, based on curing at only two temperatures, suggest that further studies at more curing rates could provide valuable insight into precise protocols to employ to minimize resistivity. It is believed that at lower temperatures and larger times there is more interaction between CNT and the matrix. Strong bonds between the two create irregularities in the CNTs that act as electron scattering sites. These scattering sites, per classic conductivity theory, reduce the net conductivity of each CNT in the composite, hence the overall conductivity.

### 4.4. Current Dependence

As observed at all extremely low loading samples (less than 0.1% wt CNT), the resistivity values herein present a current dependence, this effect was absent in the samples with loadings above 0.1% wt CNTs. This phenomenon has been previously observed by Sandler et al. [7] in a reference that seems to focus primarily on extremely low loading values. In that work, the observations are consistent with the present findings; percolation-tunneling along with excluded volumes theories are not enough to explain percolation thresholds found. Moreover, the specific conductivity of the nanocomposites in [7] presents a frequency dependence with an increase in the capacitive component with increased frequency. This mechanism is consistent with the observation that for extremely low loadings the conductivity increases with applied current/voltage. That is, increasing the applied voltage will increase the number of dielectric ‘breakdowns’ in the capacitor network of the extremely low loading samples. This will increase the net current, ergo the conductivity will increase with increasing applied voltage.

## 5. Conclusions

Detailed data on the conductivity of CNT-epoxy conductive composites fabricated using CNT bundles dispersed in epoxy resins over a wide loading range, using different dispersion and curing protocols suggests a simple heuristic model of the impact of production protocols on conductivity. First, there are two distinct regions of conductivity with a sharp drop in conductivity, about two orders of magnitude, occurring as the loading is decreased to about 0.1% wt CNT. This is consistent with percolative type conductivity for CNT loading above about 0.1% wt (low loading). As the loading increases above 0.1% wt there is a gradual increase in conductivity, anticipated for percolation-based conduction. Second, the mixing protocol is important. At any particular loading within the ‘low loading’ regime, the best conductivity is found for minimal mixing. Therefore, to create a high conductivity epoxy–CNT composite, it is best to have a CNT loading of the order 0.1% wt or higher, avoid intensive mixing of the CNTs into the matrix, and to conduct an accelerate cure at higher temperature. Much of the simple heuristic, outlined above, for the low loading samples, is consistent with theory. Based on the theory of percolation for high aspect ratio (e.g., CNT) conductive phase, percolation paths will form at low loadings. Moreover, as demonstrated using SEM, inhomogeneous microstructures, featuring excluded volumes, will form if little mixing is employed in the production protocol. As discussed in the literature, limiting the conductive phase (CNT) to a fraction of the total matrix volume, increases the local CNT density, hence increasing the percolation path density. Thus, percolation can begin at lower concentrations than for homogenous, that is well-mixed, samples.

The theory also explains behavior observed at ‘extremely low loadings’. Theory indicates that with or without mixing, a sharp drop in conductivity is anticipated at some critical conductive phase concentration, below which percolation paths cannot form. In the present work this is consistent with the sharp drop in conductivity for CNT concentrations below about 0.1% wt. Samples with extremely low CNT loading values, but which still show conductivity orders of magnitude higher than the matrix alone, which present no connectivity, require a nonpercolative model such as the dielectric breakdown observed in capacitors.
